# Genome Imputation for Genome-Wide Association Study of Reproductive Traits in Chinese Duroc, Landrace, and Yorkshire Pigs: Strategy and Validation

**DOI:** 10.3390/ani16040583

**Published:** 2026-02-12

**Authors:** Jieke Zhou, Yang Fu, Yingying Zhang, Weilong Tu, Ji Huang, Yaxu Liang, Bushe Li, Hejun Zhang, Yan Liu, Kejun Wang, Hongyang Wang, Yongsong Tan

**Affiliations:** 1 Key Laboratory of Livestock and Poultry Resources (Pig) Evaluation and Utilization, Ministry of Agriculture and Rural Affairs, Institute of Animal Husbandry & Veterinary Science, Shanghai Academy of Agricultural Sciences, Shanghai 201106, China; zhoujieke@saas.sh.cn (J.Z.); zhangyingying@saas.sh.cn (Y.Z.); tuweilong@saas.sh.cn (W.T.); android717@gmail.com (J.H.); 20250603@saas.sh.cn (Y.L.); 2 Institute of Edible Fungi, Shanghai Academy of Agricultural Sciences, National Engineering Research Center of Edible Fungi, Shanghai 201403, China; fuyang@saas.sh.cn; 3 Shanghai Engineering Research Center of Breeding Pig, Shanghai 201302, China; fordcc@163.com (B.L.); zhj19808@163.com (H.Z.); 4 Tangrenshen Group Co., Ltd., Zhuzhou 412007, China; ly9030@trsgroup.cn; 5 College of Animal Science and Technology, Henan Agricultural University, Zhengzhou 450046, China; wangkejun.me@163.com

**Keywords:** genome imputation, reproductive traits of pig, GWAS

## Abstract

Reproductive traits are critical for improving productivity and profitability in the pig industry, and genome-wide association studies (GWASs) are a powerful tool in detecting genetic markers related to target traits. Genome imputation provides an effective approach to obtain a greater number of genetic markers from low-density sequencing data. China’s pig industry recently introduced an imputation panel and is now seeking to determine what types of data are required to meet breeding needs. In this study, we collected and analyzed two pig sequencing datasets, including Yorkshire pig (YY), Landrace pig (LL), and Duroc pig (DD), genotyped by either an SNP chip (*n* = 816) or genotyping-by-targeted sequencing (*n* = 314), and applied an imputation strategy before validation in a third dataset (*n* = 2401). The aim of this study was to identify SNPs associated with reproductive traits and compare imputation results of two different types of data to evaluate whether sample size or marker density more strongly impacts imputation-enabled GWAS performance. Through a GWAS, we identified 73 significant SNPs from imputed Chip data across seven reproductive traits, 94 SNPs from imputed GBTS data across three traits, and 34 SNPs from the combined dataset across seven traits. Seven of these SNPs passed validation and were associated with number born alive, number born healthy, and gestation length. Gestation length (GL) and number born alive (NBA) are the most noteworthy traits. *LOXL2* and *PTPRD* are high-confidence candidate genes affecting GL and NBA, respectively. In addition to *LOXL2*, *STC1*, *NKX2-6*, *HMGCLL1*, *MLIP*, *TINAG*, *FAM83B*, *GFRAL*, *HCRTR2*, *ENTPD4*, *MYH8*, *IER5L*, and *U5* are associated with GL. Moreover, in addition to *PTPRD*, *KLHL32*, *U6*, *MMS22L*, and *FHL5* are associated with NBA. The results of this study indicate that sample size is of greater importance than marker density in imputation strategies and provide beneficial insights into genes affecting pigs’ reproductive traits.

## Simple Summary

Genome imputation provides an effective approach to performing GWAS with high accuracy and efficiency at acceptable cost. In this study, we used sequencing data genotyped by an SNP chip and genotyped-by-targeted sequencing for imputation, and then performed GWAS across eight reproductive traits and validated them in a third dataset. Our results indicated that sample size is of greater importance than marker density in genome imputation; we identified 197 SNPs associated with reproductive traits and 7 of them passed validation. Two high-confidence candidate genes, *LOLX2* and *PTPRD,* were identified as being associated with gestation length and number of born alive piglets. These findings provide beneficial guidance for molecular breeding efforts in China’s pig industry.

## 1. Introduction

Reproductive traits are key economic traits in the pig industry. Despite their importance, their improvement has been hindered by their low-to-moderate heritability [1]. Over the past few decades, molecular breeding methods such as marker-assisted selection (MAS) and genome selection (GS) have made it possible to effectively improve pigs’ reproductive performance [2]. High-throughput sequencing and genome-wide association studies (GWASs) have expanded the capacity to locate key variants that influence complex traits in both humans and animals. In pigs, GWASs have enabled the identification of numerous quantitative trait loci (QTL) associated with economic traits such as body size [3], carcass traits [4], meat quality [5,6], feed conversion ratio [7,8,9,10], semen-related traits [11,12], coat color [13,14], and disease resistance [15,16]. GWASs on the reproductive traits of pigs are abundant, with 7672 related QTL recorded in public databases (PigQTLdb, release 57, https://www.animalgenome.org/cgi-bin/QTLdb/SS/index, accessed on 26 August 2025).

Despite the aforementioned advantages, the power of GWASs critically depends on marker density and sample size. For the majority of Chinese pig farms, low-density methods such as SNP microarrays remain the most widely employed approach to perform GWASs under cost constraints, limiting their maximal utility. Genome imputation provides an effective means of obtaining high-density genetic markers from low-density data. Zhejiang University launched a pig haplotype reference panel for genotype imputation (PHARP, https://alphaindex.zju.edu.cn/PHARP/index.php/, accessed on 28 August 2025) in 2022 [17]. Following two years of refinement, it provides a highly reliable reference panel with whole-genome sequence data, including 6 449 individuals from 157 pig breeds, 50.3 million SNPs, and 5.8 million indels of autosomes (accessed in September 2025), achieving concordance rates greater than 0.99 (CR > 0.99) and correlation coefficients greater than 0.98 (R^2^ > 0.98). PHARP offers a powerful tool for imputation strategies in Chinese pig breeding, including both commercial lines and local breeds. However, questions remain as to whether an imputed low-density chip could reach a sufficient level of efficiency when performing GWASs with a large sample (>800 individuals) or whether higher-density data methods, such as genotyping-by-targeting sequencing, are still required; in such cases, a large sample is unfeasible due to cost considerations. Answering the above questions is vital for China’s pig industry because SNP chips stand as the most economical option. In this study, we aim to provide an insight into possible solutions by comparing the GWAS results of an imputed SNP chip and GBTS data.

As part of this study, we obtained three sequencing datasets: one genotyped by an SNP chip (Chip data) with a larger sample size (1064), one genotyped-by-targeted sequencing (GBTS) dataset with a smaller sample size (453), and a validation dataset containing 2401 samples. Ultimately, eight reproductive traits were recorded. After obtaining GWAS results, significant SNPs were validated using the analysis of variance (ANOVA) method in the validation datasets. The aim of this study is to establish whether sample size or sequencing depth has a greater influence on imputation and to determine key variants associated with reproductive traits.

## 2. Materials and Methods

### 2.1. Ethical Statement

All tissue collection procedures and animal experiments were conducted in accordance with Laboratory Animal Care and Use Guidelines approved by the Ethics Committee of Shanghai Academy of Agricultural Sciences (SAASPZ0524108).

### 2.2. Animals and Phenotypes

In this study, we collected three sequencing datasets and phenotype records of eight reproductive traits. Pigs included in the Chip data and GBTS data are raised at the core breeding farm of Tangrenshen Co., Ltd., Zhuzhou, China, under the same management practices, feed protocols, and environmental conditions. The eight reproductive traits assessed include total number of born (TNB), number of born alive (NBA), number of born healthy (NH), number of weaning litters (NW), born nest weight (BNW), adjusted nest weight at 21 days old (ANW_21_), gestation length (GL), and weaning to estrus interval (WEI); all measures were recorded under first parity. The adjusted nest weight at 21 days was calculated using the following model:

ANW_21_ = W × (2.218-0.0811 × WD + 0.0011 × WD^2^) +NC + PC

In the model, W is the nest weight during measurement; WD is weaning days; NC is the coefficient of piglet number (see Table 1); and PC is the coefficient of parity, which is 2.8 in this study (all pigs collected in first parity). The model is derived empirically from the company’s historical production data.

The Chip data contains 1064 individuals, with 816 individuals remaining after quality control (109 Duroc, 199 Landrace, and 508 Yorkshire), and their phenotypes were recorded from March 2021 to January 2022. The GBTS data contains 434 individuals from the same group of sows, with 314 individuals remaining after quality control (90 Landrace and 224 Yorkshire), and their trait information was recorded from August 2023 to June 2025. The validation data was collected from the core breeding farm of Shanghai Xiangxin company (Shanghai, China), containing 2401 individuals (1137 Duroc, 280 Landrace, and 984 Yorkshire), and phenotype information was recorded from 2024 to 2025. The mean phenotype value of all 8 traits is presented in Table 2; more detailed records can be seen in Table S1. All individuals from the three datasets were born to the same group of sows from the same farm with identical feed and management practices, thus ensuring minimal differences in environmental conditions.

### 2.3. Sequencing Dataset and Imputation

Genomic DNA was extracted from ear tissue with the TIANamp Genomic DNA Kit (DP304). The Chip data was genotyped using KPS Porcine Breeding 50 K chip v1 (Compass Biotechnology Company, Beijing, China), including 51,315 SNPs. The GBTS dataset was produced by means of genotyped-by-targeted sequencing with an 80 K GBTS panel (Wuhan Yingzi Gene Technology Co., Ltd., Wuhan, Hubei province, China), generating 230,599 SNPs. Genome imputation was performed with the PHARP v4 panel using Beagle 5.5 software with default parameters [18]. Imputation was performed sequentially using chromosomes and then concatenated using bcftools v1.18 [19]. GWASs were performed respectively for the Chip data, GBTS data, and their combination. Post-imputation quality control was implemented in PLINK v1.9 [20], with the following thresholds: minor allele frequency (MAF) > 0.05, SNP call rate > 90%, individual call rate > 90%, and Hardy–Weinberg equilibrium (HWE) test > 1 × 10^−6^. After filtering, a total of 6,211,038 SNPs were retained from the Chip data, 10,660,274 SNPs were retained from the GBTS data, and 1,802,522 SNPs were retained from the combined data.

### 2.4. Genome-Wide Association Study

Genome-wide association analyses were conducted in R using the rMVP package [21] with the following model:

*Y* = *Xa* + *Zb* + *e*

In the model, *Y* is the phenotype value vector; *a* is the breed effect and *X* is the corresponding matrix; *b* is the SNP effect and *Z* is the corresponding matrix; and *e* is the random residual effects.

We employed the fixed and random model circulating probability unification (farmCPU) method, which exhibits greater statistical power than the general linear model (GLM) and mixed linear model (MLM) under different population structures [22]. Use of farmCPU led to improvements in the MLM method through partitioning of the MLM into two parts, fixed and random effect components, and then iterating between them to improve detection efficiency for marker–phenotype associations. The variance components estimation method was estimated using factored spectrally transformed linear mixed models (FaST-LMM), which involve singular value decomposition on the genotype matrix and improve computational efficiency and power [23]. Multiple testing involved the Bonferroni approach with suggestive significance set as *p* < 1/N and extreme significance set as *p* < 0.05/N, where N is the number of SNPs. Q-Q and Manhattan plots were additionally drawn using the rMVP package.

### 2.5. Validation of GWAS Results

To validate our GWAS results, the association between significant SNPs and target traits was tested in validation data. Only significant SNPs found by means of a GWAS were considered. Analysis of variance (ANOVA) was performed using the R function “aov” with the same model as used in the GWAS.

In addition, multiple comparison analysis was conducted with the “TukeyHSD” function. The validation model included breed and genotype at the target locus as fixed effects.

## 3. Results

### 3.1. Significant SNPs Detected from Unimputed Data

The GWAS based on the unimputed Chip dataset resulted in only five suggestively significant SNPs (*p* < 1.9 × 10^−5^) for NW and WEI; however, none of these SNPs were annotated to functional genes. GWAS based on the unimputed GBTS dataset resulted in three extremely significant SNPs, one for NH and two for WEI, and fourteen suggestively significant SNPs, one for NH, eight for BNW, one for NW, four for GL, and four for WEI (see Table 3).

### 3.2. Significant SNPs Detected from Imputed Chip Data

**Figure 1:**
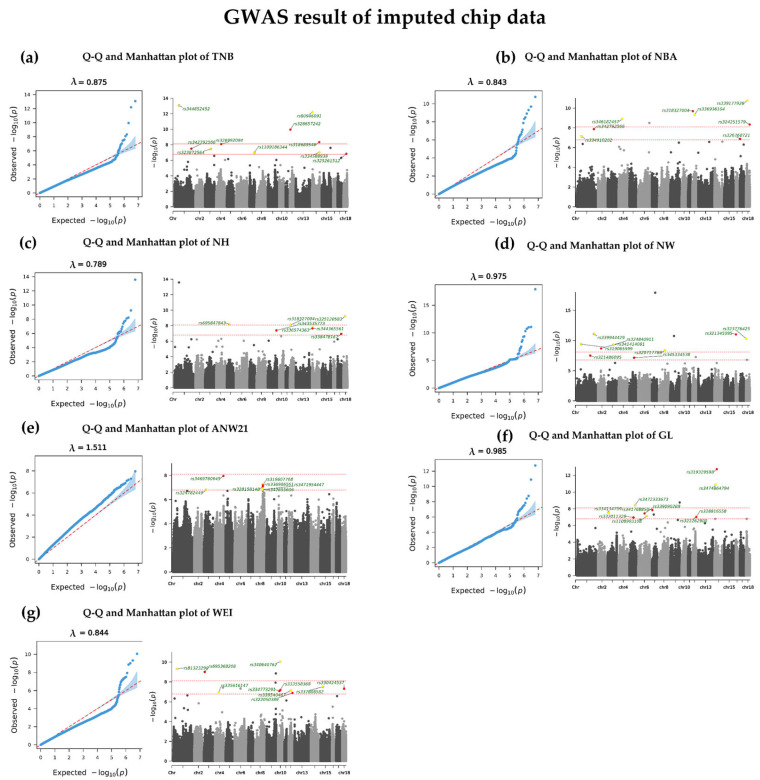
GWAS results generated using imputed Chip data. (**a**) TNB: total number born; (**b**) NBA: number born alive; (**c**) NH: number born healthy; (**d**) NW: number of weaning litters; (**e**) ANW21: adjusted nest weight at 21 days old; (**f**) GL: gestation length; (**g**) WEI: weaning to estrus interval. Red dots represent notable SNPs with annotation; the lower red line represents the suggestively significant threshold; the upper red line represents the extremely significant threshold.

After imputation with PHARP v4, the GWAS of the Chip data resulted in the identification of 73 significant SNPs, with corresponding Manhattan and Q-Q plots shown in Figure 1. Thirty of these SNPs are extremely significant (*p* < 8.05 × 10^−9^) across six traits: TNB (*n* = 4), NBA (*n* = 6), NH (*n* = 4), NW (*n* = 9), GL (*n* = 4), and WEI (*n* = 3) (see Table 4). Forty-three SNPs are suggestively significant (*p* < 1.61 × 10^−7^) across seven traits: TNB (*n* = 7), NBA (*n* = 3), NH (*n* = 4), NW (*n* = 3), ANW_21_ (*n* = 7), GL (*n* = 8), and WEI (*n* = 11) (see Table S2). In total, 479 genes were annotated, among which 456 are the protein-coding biotype based on Ensemble.

### 3.3. Significant SNPs Detected from Imputed GBTS Data

**Figure 2:**
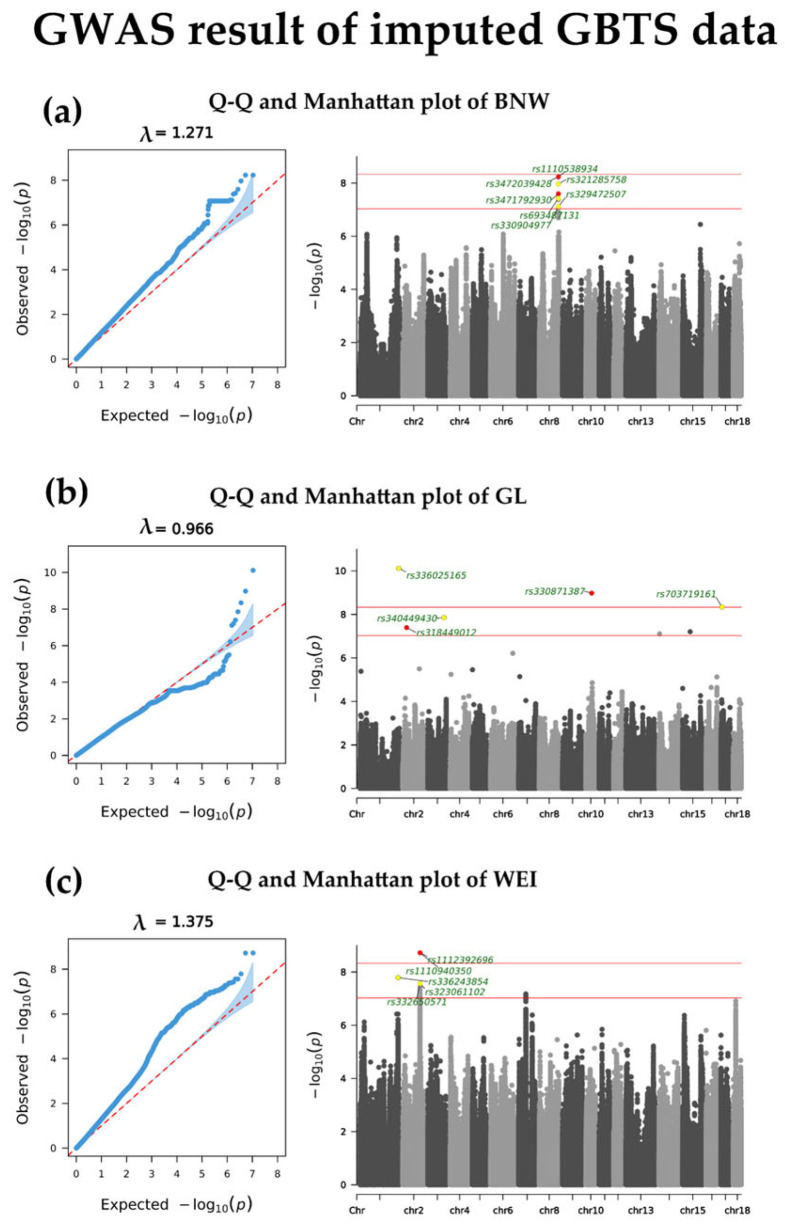
QQ and Manhattan plots of GWAS results using imputed Chip data: (**a**) BNW: birth nest weight; (**b**) GL: gestation length; (**c**) WEI: weaning to estrus interval. Red dots represent notable SNPs with annotation; the lower red line represents the suggestively significant threshold; the upper red line represents the extremely significant threshold.

The GWAS based on GBTS data resulted in the detection of significant SNPs across only BNW, GL, and WEI, with corresponding Manhattan and Q-Q plots shown in Figure 2. Five of these SNPs are extremely significant (*p* < 4.69 × 10^−9^), three for GL and two for WEI (see Table 5). In addition, 89 are suggestively significant (*p* < 9.38 × 10^−8^), 54 for TNB, 4 for GL, and 31 for WEI (see Table S3). In total, 116 genes were annotated, 3 for TNB, 87 for GL, and 26 for WEI, and 112 of them are the protein-coding type.

### 3.4. Significant SNPs Detected from Combined Imputed Data

**Figure 3:**
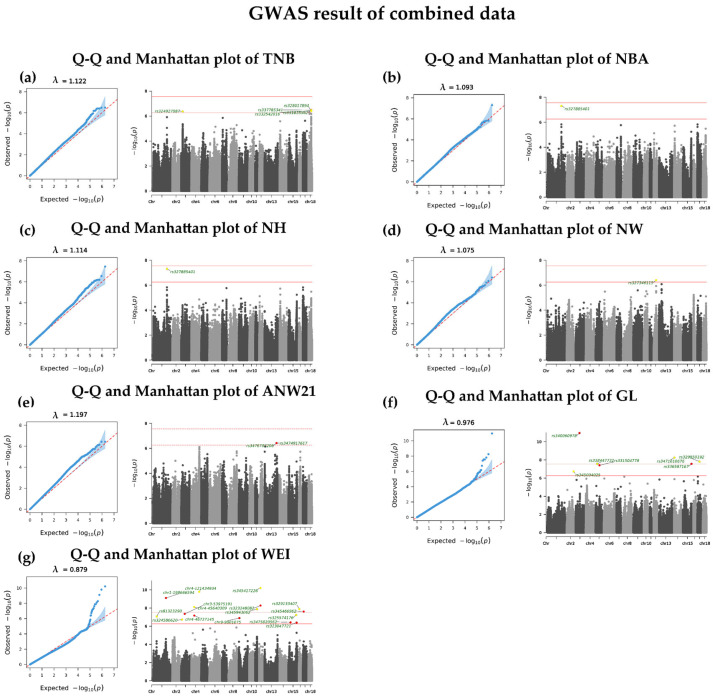
QQ and Manhattan plots of GWAS results using combined data: (**a**) TNB: total number born; (**b**) NBA: number born alive; (**c**) NH: number born healthy; (**d**) NW: number of weaning litters; (**e**) ANW21: adjusted nest weight at 21 days old; (**f**) GL: gestation length; (**g**) WEI: weaning to estrus interval. Red dots represent notable SNPs with annotation; the lower red line represents the suggestively significant threshold; the upper red line represents the extremely significant threshold.

In the combined data of the imputed Chip and GBTS data, 34 significant SNPs were detected (see Figure 3). Thirteen of these SNPs are extremely significant (*p* < 2.77 × 10^−8^) (see Table 6), five for GL and eight for WEI (see Table 6). The other 21 suggestively significant SNPs (*p* < 5.54 × 10^−7^) include 5 SNPs for TNB, 1 for NBA, 2 for NH, 1 for NW, 2 for ANW_21_, 2 for GL, and 8 for WEI (see Table S4). The only SNP that overlapped with the imputed Chip data was rs81323290 (on chromosome 1, 57,377,646 bp position, see Tables S2 and S4), associated with WEI. Two SNPs are not only associated with TNB but also NH: rs327885401 (on chromosome 1, 213,444,403 bp position) and rs337785341 (on chromosome 18, 42,955,750 bp position).

### 3.5. Validation of Significant SNPs in the Third Dataset

All 198 significant SNPs (in the three datasets, 201 SNPs were identified; however, three of them were repeated), both suggestively and extremely, were validated via ANOVA with the same model as applied in the GWAS.

After analysis, seven SNPs passed the significant thresholds (*p* < 0.05) (see Table 7), including four from imputed Chip data across three traits: NBA (*n* = 1), NH (*n* = 1), and GL (*n* = 2); one from imputed GBTS data on GL; and two from combined imputed data, one for NBA and one for GL. Genotype–phenotype summaries are presented in Table 8. Overall, advantageous genotypes for GL include CC, GG, CT, and AG at rs344603744, rs328816558, rs336025165, and rs3471610676, respectively; advantageous genotypes for NBA include AA and AA at rs334910202 and rs327885401, respectively; and the advantageous genotype for NH is CT at rs344365561.

### 3.6. Comparison of Imputed Chip and GBTS Data Performance

The GWAS performance of all datasets used in this study is summarized in Table 9. The use of both Chip and GBTS data resulted in the identification of more SNPs after imputation. Chip data led to the identification of more associated reproductive traits after imputation (from two to seven); however, GBTS data resulted in the identification of fewer associated traits after imputation (from five to three); in addition, it was marked by the lowest pass rate during validation. Although combined data is marked by the highest pass rate during validation, it identified the lowest number of SNPs, and its validation rate was not significantly higher than that of the imputed Chip data. Overall, imputed Chip data exhibited the best performance in the GWAS.

## 4. Discussion

Reproductive traits are the primary economic traits for the pig industry, with genome imputation enabling widespread use of GWASs for China’s pig industry following the release of the PHARP panel in 2022 [24,25,26,27]. Despite these advances, little is known about the factors that determine accuracy and efficiency in genome imputation, whether they are determined by sample size or sequencing density. In this study, we used low-density Chip data with a larger sample size (814) and high-density GBTS data with a smaller sample size (314) to perform genome imputation and subsequent genome-wide association analysis. All 198 significant SNPs were validated in a third dataset.

Our results provide conclusive evidence that imputed data provide greater efficiency than ever before, with both Chip data and GBTS data identifying a greater number of significant SNPs after imputation. Overall, Chip data showed greater efficiency than GBTS data. The number of identified SNPs was close (73 versus 94); however, imputed Chip data contains an evidently greater number of extremely significant SNPs than imputed GBTS data (30 versus 13), with more SNPs passing validation (four versus one). Combined data outperformed GBTS data but did not surpass Chip data. Our results also raised further questions with regard to imputation strategy. Imputed Chip data exhibited a small genomic inflation factor, suggesting that low-density data may hinder detection power when performing GWAS; in comparison, imputed GBTS data showed a too high genomic inflation factor, suggesting that a small sample size may result in a higher false positive rate. Their combination led to improvements but resulted in the loss of a significant number of SNPs after quality control and minimal gain in higher detection efficiency. Overall, under cost constraints, large-sample sequencing using an SNP chip is sufficient to perform more efficient GWASs after imputation in this study design and populations.

GL harbors the greatest number of associated SNPs: rs344603744, rs328816558, rs336025165, and rs3471610676. Within the 500 kb region upstream and downstream of these loci, a total of 25 protein-coding candidate genes were annotated. Among them, the *LOXL2* gene carries an intron variant (rs3471610676), making it a strong candidate gene for GL. The variant of rs3471610676 is A/G, and its advantageous genotype is AG in Duroc (1.16 days shorter) and Landrace (0.3 days shorter), and AA in Yorkshire pigs (0.4 days shorter). Although *LOXL2* is not reported to have a direct association with pigs’ GL, evidence has demonstrated its high expression in the reproductive tissue of humans, mice, and pigs [28,29,30], particularly in the placenta, uterus, and prostate [28]. Yang [30] compared Duroc and Meishan pigs, an indigenous Chinese breed renowned for its excellent reproductive performance, and observed higher *LOXL2* expression levels in the Meishan pigs’ reproductive tissue, which may enhance endometrial angiogenesis. In another study on pigs, researchers reported differential *LOXL2* expression in muscle tissue between Mali and Hampshire breeds in Assam, India [31]. The authors of a study on human preterm prelabor rupture (pPROM) found that *LOXL2* is more significantly expressed in fetal membranes from pPROM [29], particularly in the ruptured temporal artery. In a recent study, researchers found that lysyl oxidases (encoded by the *LOXL* gene family) are necessary for on-time parturition in mice [32]. Based on the results of these studies, Lysyl oxidases play an important role in maintaining the integrity of the myometrial extracellular matrix (ECM). Inhibiting lysyl oxidases will result in myometrial dysfunction through alteration of ECM composition and the subsequent delay of parturition [32]. The same mechanism may be applied to pigs’ gestation length. In addition to *LOXL2*, 12 other genes are reported to be associated with reproductive traits [24,33]: *STC1*, *NKX2-6*, *HMGCLL1*, *MLIP*, *TINAG*, *FAM83B*, *GFRAL*, *HCRTR2*, *ENTPD4*, *MYH8*, *IER5L,* and *U5*. Of these genes, three map with rs3471610676 (*STC1*, *NKX2-6,* and *ENTPD4*), six map with rs344603744 (*HMGCLL1*, *MLIP*, *TINAG*, *FAM83B*, *GFRAL*, and *HCRTR2*), one maps with rs328816558 (*MYH8*), and the last two map with rs336025165 (*IER5L* and *U5*). These relationships provide a set of high-confidence candidate genes for GL.

In early 2004, Danmark introduced litter size at day 5 after farrowing (LS5) into pig breeding [34]. LS5 proved to be an efficient indicator of reproductive performance, with greater performance than TNB alone. In our study, the NBA trait possesses a noticeable associated gene, *PTPRD*, and its AA genotype shows a clear advantage in all three breeds: 1.67 for Duroc, 1.86 for LL, and 0.88 for YY (see Table 8). The connection between *PTPRD* and pigs’ meat quality has long been recognized [35,36], with two variants in *PTPRD* being significantly associated with pigs’ fat thickness and muscle firmness [35]. In another study, researchers performed a GWAS and identified *PTPRD* within a QTL related to pigs’ carcass traits [36]. In a recent study, *PTPRD* was identified as a candidate gene associated with pigs’ reproductive traits [37]. Beyond pigs, evidence indicates that *PTPRD* may cause human trigonocephaly, hearing loss, and intellectual disability [38]. *PTPRD* also plays a functional role in the nervous system based on studies performed on humans and mice; in one study, it was found to participate in embryonic neurogenesis and may contribute to neurological disorders such as restless legs syndrome [39], Alzheimer’s disease, attention-deficit/hyperactivity disorder, obsessive–compulsive disorder, and autism spectrum disorder [40]. Another variant in *PTPRD* is significantly associated with hypertension diagnosis [41]. Evidence indicates that *PTPRD* plays a vital role in neuronal cell adhesion molecules and synaptic specifiers, indicating that its variant can cause the nervous system diseases described above. Its malfunction may also cause congenital fatal diseases in pigs [42]. Together, the above evidence strongly suggests *PTPRD* as a potential key gene influencing piglets’ survival. Other novel genes associated with NBA found in our study, excluding *PTPRD,* include *KLHL32*, *U6*, *MMS22L,* and *FHL5*; how these genes specifically influence NBA requires further investigation.

## 5. Conclusions

In conclusion, in this study, we collected two sequencing datasets: SNP chip data is a low-density dataset with a larger sample size, whereas GBTS data is a high-density dataset with a smaller sample size. Both datasets were imputed with the PHARP panel before the GWAS was performed to determine loci associated with eight reproductive traits, TNB, NBA, NH, NW, BNW, ANW_21_, GL, and WEI, and establish whether sample size or marker density more strongly impacts the performance of GWASs after imputation. Our results show that imputed Chip data performed better than GBTS with lower density markers but a larger sample size, suggesting that a larger sample is of greater importance than marker density in genome imputation in this study design and populations. Our GWAS resulted in the detection of 198 unique significant SNPs (73 from imputed Chip data, 94 from imputed GBTS, and 34 unique to combined data, of which 3 SNPs were repeated) associated with reproductive traits, with GL and NBA being the most noteworthy traits. *LOXL2* and *PTPRD* are high-confidence candidate genes associated with GL and NBA. In this study, we detected novel genes related to pigs’ reproductive traits that will be of practical significance for molecular breeding efforts in China’s pig industry.

## Figures and Tables

**Table 1:** Coefficient of piglet number when calculating ANW21.

Piglet Number	Coefficient
<3	47.2
3	34.5
4	27.7
5	23.2
6	18.6
7	13.6
8	9.5
>8	7.7

**Table 2:** Mean phenotype value of all 8 reproductive traits.

Data Source	Trait	Breed		
		DD	LL	YY
Chip data	TNB ^1^	9.19	13.73	15.16
	NBA ^2^	7.7	12.67	13.97
	NH ^3^	7.57	12.5	13.73
	NW ^4^	7.08	11.92	12.17
	BNW ^5^/kg	12.14	19.56	19.24
	ANW21 ^6^/kg	55.95	66.08	63.07
	GL ^7^/day	117.41	118.88	118.05
	WEI ^8^/day	15.6	6.48	9.04
GBTS	TNB	-	15.5	16.57
	NBA	-	13.6	14.41
	NH	-	13.11	13.71
	NW	-	12.89	13.13
	BNW/kg	-	17.72	18.34
	ANW21/kg	-	69.64	70.43
	GL/day	-	118.39	117.8
	WEI/day	-	2.96	3.02

^1^ TNB: total number born; ^2^ NBA: number born alive; ^3^ NH: number born healthy; ^4^ NW: number of weaning litters; ^5^ BNW: birth nest weight; ^6^ ANW21: adjusted nest weight at 21 days old; ^7^ GL: gestation length; ^8^ WEI: weaning to estrus interval.

**Table 3:** Significant SNPs detected via the GWAS with unimputed data.

Traits	Data	SNP	Chr	Position	*P*	Associated Genes
NH ^1^	GBTS data	rs1107471812	5	63,649,651	1.95 × 10^−7^	*ATN1*, *CD163*, *NCAPD2*, *CDCA3*, *CLSTN3*
		rs343985646	5	63,712,118	1.54 × 10^−6^	*ATN1*, *CD163*, *NCAPD2*, *CDCA3*, *CLSTN3*
NW ^2^	Chip data	CNC10013394	1	191,110,440	5.34 × 10^−7^	*HIF1A*, *ISG20*, *MRPS11*, *SYT16*, *NTRK3*
		CNC10023206	2	156,359,053	2.93 × 10^−6^	*-*
		CNC10051796	5	95,090,653	1.46 × 10^−7^	*-*
		CNC10091386	9	69,719,963	5.34 × 10^−7^	*STEAP1*
		CNC10120577	12	27,847,549	5.34 × 10^−7^	*CA10*, *NME1*, *NME2*, *MBTD1*, *SPAG9*
	GBTS data	rs320174932	5	94,697,959	1.08 × 10^−6^	*RLIG1*,*TMTC3*
WEI ^3^	Chip data	CNC10013394	1	191,110,440	8.42 × 10^−7^	*HIF1A*, *ISG20*, *MRPS11*, *SYT16*, *NTRK3*
		CNC10051796	5	95,090,653	6.09 × 10^−7^	*-*
		CNC10091386	9	69,719,963	8.42 × 10^−7^	*STEAP1*
		CNC10120577	12	27,847,549	8.42 × 10^−7^	*CA10*, *NME1*, *NME2*, *MBTD1*, *SPAG9*
		CNC10130159	13	8,011,066	2.80 × 10^−7^	*ZNF385D*
	GBTS data	rs340665731	18	17,335,467	1.78 × 10^−6^	*MKLN1*, *PLXNA4*
		rs81472220	18	17,378,156	2.93 × 10^−7^	*MKLN1*, *PLXNA4*
		rs339243967	18	17,378,165	2.93 × 10^−7^	*MKLN1*, *PLXNA4*
		rs322371592	18	17,378,186	7.62 × 10^−7^	*MKLN1*, *PLXNA4*
		rs331818943	18	17,378,193	1.43 × 10^−6^	*MKLN1*, *PLXNA4*
BNW ^4^	GBTS data	rs330024119	5	60,237,794	4.99 × 10^−7^	*BCL2L14*, *DUSP16*, *GPR19*, *MANSC1*
		rs690225344	5	60,274,281	4.99 × 10^−7^	*BCL2L14*, *DUSP16*, *GPR19*, *MANSC1*
		rs81384448	5	60,402,824	4.99 × 10^−7^	*BCL2L14*, *DUSP16*, *GPR19*, *MANSC1*
GL ^5^	GBTS data	rs323307717	17	7,366,047	1.66 × 10^−6^	*TRIML1*, *TRIML2*
		rs334321909	17	7,366,141	9.14 × 10^−7^	*TRIML1*, *TRIML2*
		rs340297296	17	7,557,349	5.70 × 10^−7^	*TRIML1*, *TRIML2*
		rs321575957	17	7,636,108	7.65 × 10^−7^	*TRIML1*, *TRIML2*

^1^ NH: number born healthy; ^2^ NW: number of weaning litters; ^3^ WEI: weaning to estrus interval; ^4^ BNW: birth nest weight; ^5^ GL: gestation length.

**Table 4:** Extremely significant SNPs detected via the GWAS with imputed Chip data.

Trait	Chr	Position	*P*	Associated Genes
TNB ^1^	1	63,898,997	8.43 × 10^−14^	*FHL5*, *GPR63*, *MMS22L*, *NDUFAF4*, *KLHL32*
	12	7,855,277	1.12 × 10^−10^	*CDC42EP4*, *U2*, *U6*, *VCF1*, *SLC39A11*, *MTNAP1*, *SSTR2*
	14	6,507,547	6.34 × 10^−13^	*BMP1*, *DMTN*, *EGR3*, *PEBP4*, *SFTPC*, *LGI3*, *SLC39A14*, *REEP4*, *SORBS3*
	14	101,649,892	4.63 × 10^−9^	*IFIT1*, *KIF20B*, *LIPA*, *PANK1*, *SLC16A12*
NBA ^2^	4	51,290,366	1.22 × 10^−9^	*CA13*, *E2F5*, *LRRCC1*, *RALYL*, *CA1*, *CA2*, *CA3*, *RBIS*
	6	154,696,765	3.17 × 10^−9^	*DAB1*
	12	7,980,548	2.07 × 10^−10^	*CDC42EP4*, *U6*, *VCF1*, *SLC39A11*
	12	32,517,221	4.87 × 10^−10^	*ANKFN1*,
	18	19,982,267	1.71 × 10^−11^	*KCP*, *LEP*, *OPN1SW*, *SND1*, *TNPO3*, *IMPDH1*, *CALU*, *CCDC136*, *FLNC*, *GARIN1A*, *IRF5*, *LRRC4*, *PRRT4*, *RBM28*, *SPMIP1*
	18	52,578,093	4.62 × 10^−9^	*INHBA*, *GLI3*
NH ^3^	1	63,897,503	2.66 × 10^−14^	*FHL5*, *GPR63*, *MMS22L*, *NDUFAF4*, *UFL1*, *KLHL32*
	5	31,319,347	5.95 × 10^−9^	*U6*, *CAND1*, *GRIP1*
	12	7,980,548	8.04 × 10^−9^	*CDC42EP4*, *U6*, *VCF1*, *SLC39A11*, *COG1*, *MTNAP1*, *SSTR2*, *U2*
	18	25,101,493	5.79 × 10^−10^	*AASS*, *CAGLS2*, *FEZF1*, *PTPRZ1*, *FAM3C*
NW ^4^	1	56,604,908	4.11 × 10^−10^	*ALDH1B1*, *TSTD2*, *XPA*, *CCDC180*, *IGFBPL1*, *NCBP1*, *SHB*, *TDRD7*, *TMOD1*
	1	239,142,635	8.30 × 10^−12^	*RNGTT*, *SPACA1*, *CNR1*, *U1*
	2	44,031,802	2.11 × 10^−9^	*PDE3B*, *U6*, *CYP2R1*, *INSC*
	3	85,218,715	5.22 × 10^−10^	*CFAP36*, *CCDC85A*, *EFEMP1*, *PNPT1*, *PPP4R3B*
	7	46,295,972	1.20 × 10^−18^	*-*
	8	86,270,554	3.99 × 10^−9^	*ELMOD2*, *SCOC*, *RNF150*, *CLGN*, *MGAT4D*, *TBC1D9*, *ZNF330*
	9	53,533,517	1.79 × 10^−11^	*DCPS*, *DDX25*, *FAM118B*, *FOXRED1*, *ST3GAL4*, *TIRAP*, *VSIG10L2*, *KIRREL3*, *CDON*, *RPUSD4*, *SRPRA*
	16	4,299,131	9.94 × 10^−12^	*OTULIN*, *OTULINL*, *TRIO*, *U6*, *ANKH*, *FBXL7*
	18	1,995,040	4.77 × 10^−11^	*LMBR1*, *MNX1*, *DNAJB6*, *NOM1*, *UBE3C*
GL ^5^	5	81,100,973	3.85 × 10^−9^	*ASCL1*, *NT5DC3*, *MODIFIER*, *PAH*, *U6*
	9	132,612,099	1.76 × 10^−9^	*KCNH1*, *SERTAD4*, *SYT14*, *UTP25*, *HHAT*, *U5*
	14	15,733,425	1.26 × 10^−11^	*CEP44*, *GLRA3*, *HPGD*, *ADAM29*, *FBXO8*
	14	31,997,523	1.82 × 10^−13^	*ATP2A2*, *FAM216A*, *HVCN1*, *IFT81*, *MYL2*, *PPP1CC*, *RAD9B*, *TCTN1*, *ANAPC7*, *ARPC3*, *CCDC63*, *CUX2*, *GPN3*, *PPTC7*, *VPS29*
WEI ^6^	1	57,377,646	4.83 × 10^−10^	*LYRM2*, *MDN1*, *PM20D2*, *PNRC1*, *RRAGD*, *ANKRD6*, *CASP8AP2*, *GABRR1*, *GABRR2*, *GJA10*, *RNGTT*, *SRSF12*, *U6*, *UBE2J1*
	3	11,951,819	9.87 × 10^−10^	*CASTOR2*, *GTF2I*, *GTF2IRD1*, *NCF1*, *RCC1L*
	9	131,092,632	1.40 × 10^−9^	*BATF3*, *DTL*, *NSL1*, *PACC1*, *SPATA45*, *U6*, *ATF3*, *INTS7*, *LPGAT1*, *NEK2*, *NENF*, *PPP2R5A*, *TATDN3*
	10	29,606,662	8.91 × 10^−11^	*AGTPBP1*, *GOLM1*, *NTRK2*

^1^ TNB: total number born; ^2^ NBA: number born alive; ^3^ NH: number born healthy; ^4^ NW: number of weaning litters; ^5^ GL: gestation length; ^6^ WEI: weaning to estrus interval.

**Table 5:** Extremely significant SNPs detected via the GWAS with imputed GBTS data.

Trait	Chr	Position	*P*	Associated Genes
GL ^1^	1	269,242,563	7.66 × 10^−11^	*SLC27A4*, *URM1*, *SPTAN1*, *DYNC2I2*, *PKN3*
	10	41,133,816	1.05 × 10^−9^	*MTPAP*, *JCAD*, *SVIL*, *MAP3K8*, *ZNF438*
	17	7,612,732	4.56 × 10^−9^	*TRIML2*, *TRIML1*
WEI ^2^	2	119,984,294	1.89 × 10^−9^	*FEM1C*, *LVRN*, *COMMD10*, *TMED7*, *ATG12*, *ARL14EPL*, *CDO1*,
	2	119,984,458	1.87 × 10^−9^	*FEM1C*, *LVRN*, *COMMD10*, *TMED7*, *ATG12*, *ARL14EPL*, *CDO1*

^1^ GL: gestation length; ^2^ WEI: weaning to estrus interval.

**Table 6:** Extremely significant SNPs detected via the GWAS with imputed combined data.

Trait	Chr	Position	*P*	Associated Genes
GL ^1^	3	59,044,693	1.10 × 10^−11^	*PTCD3*, *POLR1A*, *ST3GAL5*, *MAT2A*, *SFTPB*, *ELMOD3*, *CAPG*, *VAMP5*
	5	25,700,891	2.74 × 10^−8^	-
	14	7,472,833	5.61 × 10^−9^	*PEBP4*, *RHOBTB2*, *STC1*, *LOXL2*, *ENTPD4*, *CHMP7*, *PEBP4*
	15	131,809,054	2.71 × 10^−8^	*CAB39*, *ITM2C*, *SPATA3*, *PSMD1*, *NMUR1*, *ARMC9*
	17	33,041,768	1.53 × 10^−8^	*OXT*, *MRPS26*, *PTPRA*, *VPS16*, *PCED1A*, *TMEM239*, *CPXM1*
WEI ^2^	1	198,666,594	7.80 × 10^−10^	*IZUMO3*, *U6*, *ELAVL2*
	4	45,640,309	7.77 × 10^−9^	*OTUD6B*, *PIP4P2*, *NECAB1*, *LRRC69*, *SLC26A7*
	4	121,434,934	1.62 × 10^−10^	*KRT39*, *CCR7*, *TNS4*, *IGFBP4*, *TOP2A*, *SMARCE1*, *NR1D1*, *KRTAP3-1*
	11	27,834,915	1.3 × 10^−8^	-
	12	21,385,205	5.91x10^−9^	*HAP1*, *JUP*, *P3H4*, *EIF1*, *CCR7*, *GAST*, *U6*, *SMARCE1*
	12	21,886,785	6.14 × 10^−11^	*PTBP2*, *U6*
	16	6,390,533	1.31 × 10^−8^	*U6*, *MYO10*
	17	12,211,801	2.45 × 10^−8^	*U6*, *CHRNA6*, *CHRNB3*, *HOOK3*, *POMK*, *RNF170*, *THAP1*, *FNTA*, *CSGALNACT1*

^1^ GL: gestation length; ^2^ WEI: weaning to estrus interval.

**Table 7:** Significant SNPs after ANOVA with the validation dataset.

Data Source	Trait	SNP	Chr	POS	*p*	Associated Genes
Chip data	NBA ^1^	rs334910202	1	64,152,697	0.015	*MMS22L*, *KLHL32*,
	NH ^2^	rs344365561	14	5,800,493	0.008	*GFRA2*
	GL ^3^	rs344603744	7	26,188,159	0.016	*HMGCLL1*, *MLIP*, *TINAG*, *FAM83B*, *GFRAL*, *HCRTR2*
		rs328816558	12	55,594,892	0.022	*ADPRM*, *DNAH9*, *MYH8*, *SCO1*
GBTS	GL	rs336025165	1	269,242,563	0.015	*IER5L*, *LRRC8A*, *U5*
Combined data	NBA	rs327885401	1	213,444,403	0.037	*PTPRD*
	GL	rs3471610676	14	7,472,833	0.024	*LOXL2*, *STC1*, *NKX2-6*, *ENTPD4*

^1^NBA: number born alive; ^2^ NH: number born healthy; ^3^ GL: gestation length.

**Table 8:** Genotype–phenotype summaries.

Trait	SNP	Breed	Phenotype Value of Genotype		
			CC	CT	TT
GL ^2^	rs344603744	DD	115.24 ± 0.19 ^a^	115.3 ± 0.06 ^b^	115.36 ± 0.05 ^b^
		LL	116.03 ± 0.19 ^a^	116.66 ± 0.12 ^b^	116.57 ± 0.13 ^b^
		YY	114.55 ± 0.39 ^a^	115.31 ± 0.09 ^b^	115.17 ± 0.05 ^b^
			**AA**	**AG**	**GG**
	rs328816558	DD	115.35 ± 0.04 ^b^	115.24 ± 0.09 ^a^	115.57 ± 0.3 ^b^
		LL	116.53 ± 0.1 ^a^	116.62 ± 0.14 ^a^	116 ± 0.45 ^b^
		YY	115.31 ± 0.05 ^a^	115.04 ± 0.07 ^b^	114.93 ± 0.14 ^b^
			**CC**	**CT**	**TT**
	rs336025165	DD	115.59 ± 0.04 ^a^	115.35 ± 0.08 ^b^	115.8 ± 0.39 ^a^
		LL	116.5 ± 0.22 ^a^	116.54 ± 0.14 ^a^	116.54 ± 0.11 ^a^
		YY	115.48 ± 0.09 ^a^	115.22 ± 0.06 ^b^	115.25 ± 0.1 ^b^
			**AA**	**AG**	**GG**
	rs3471610676	DD	-	114 ± 0 ^a^	115.16 ± 0.08 ^b^
		LL	116.86 ± 0.35 ^a^	116.39 ± 0.11 ^b^	116.61 ± 0.12 ^c^
		YY	114.88 ± 0.44 ^a^	115.01 ± 0.09 ^b^	115.24 ± 0.05 ^b^
			**AA**	**AG**	**GG**
NBA ^3^	rs334910202	DD	-	-	7.83
		LL	11.1 ± 0.29 ^a^	10.74 ± 0.23 ^b^	11.76 ± 0.27 ^a^
		YY	11.76 ± 0.39 ^a^	11 ± 0.19 ^b^	11.28 ± 0.2 ^a^
			**AA**	**AG**	**GG**
	rs327885401	DD	9.5 ± 0.87 ^a^	7.75 ± 0.19 ^b^	7.83 ± 0.08 ^b^
		LL	13 ± 0 ^a^	10.04 ± 0.5 ^b^	11.14 ± 0.17 ^b^
		YY	12.03 ± 0.42 ^a^	11.38 ± 0.15 ^b^	11.15 ± 0.14 ^b^
			**CC**	**CT**	**TT**
NH ^4^	rs344365561	DD	8.2 ± 0.66 ^a^	8.39 ± 0.21 ^a^	7.97 ± 0.17 ^b^
		LL	10.5 ± 1.19 ^a^	11.05 ± 0.16 ^b^	10.71 ± 1.78 ^a^
		YY	10.55 ± 0.32 ^a^	11.34 ± 0.14 ^b^	11.28 ± 0.2 ^b^

Identical uppercase letters indicate no difference and different uppercase letters indicate a significant difference. ^2^ GL: gestation length; ^3^ NBA: number born alive; ^4^ NH: number born healthy.

**Table 9:** Summary of the GWAS efficiency of the three datasets.

Dataset	Number of All Significant SNPs		Number of Extremely Significant SNPs and Rate		Number of SNPs Passed Validation and Rate		Number of Associated Traits	
	Unimputed	Imputed	Unimputed	Imputed	Unimputed	Imputed	Unimputed	Imputed
Chip	5	73	0	30 (41%)	-	4 (4.3%)	2	7
GBTS	17	94	3	5 (5%)	-	1 (1.1%)	5	3
Combined data	-	34	-	13 (38%)	-	2 (5.9%)	-	7

## Data Availability

The datasets generated and/or analyzed during the current study are available from the corresponding author upon reasonable request.

## Supplementary Materials

The following supporting information can be downloaded at https://www.mdpi.com/article/10.3390/ani16040583/s1. Table S1: Phenotype records of reproductive traits; Table S2: Identified SNPs in imputed Chip data; Table S3: Identified SNPs in imputed GBTS data; Table S4: Identified SNPs in combined imputed data.

## Abbreviations

The following abbreviations are used in this manuscript:

TNB	Total born number
NBA	Number born alive
NH	Number born healthy
NW	Number of weaning litters
BNW	Born nest weight
ANW_21_	Adjusted nest weight at 21 days
GL	Gestation length
WEI	Weaning to estrus interval
